# Traffic flow prediction based on temporal attention and multi-graph adjacency fusion using DynamicChebNet

**DOI:** 10.1038/s41598-025-12598-z

**Published:** 2025-10-07

**Authors:** Jingbao Zhang, Junbing Cheng, Fujia Li

**Affiliations:** 1 College of Artificial Intelligence, Taiyuan University of Technology, Taiyuan, 030024 Shanxi China; 2 Present Address: School of Computer Science and Engineering, Tianjin University of Technology, 300384, Tianjin, China

**Keywords:** Traffic Flow Prediction, Temporal Attention, Dynamic ChebNet, Multi-Graph Adjacency Matrix Fusion, Spatiotemporal Dependencies, Applied mathematics, Computer science

## Abstract

Accurate and timely traffic flow prediction plays a crucial role in improving road utilization, reducing congestion, and optimizing public transportation management. However, modern urban traffic faces challenges such as complex road network structures and the variation in traffic flow across different temporal and spatial scales. These issues lead to complex spatiotemporal correlations and heterogeneity, resulting in low prediction accuracy and poor real-time performance of existing models. In this work, we propose a novel traffic flow prediction model called TMDCN (Temporal Attention and Multi-Graph Adjacency Fusion Using DynamicChebNet), which integrates temporal attention and multi-graph adjacency matrix fusion. First, to address the difficulty of capturing dependencies across multiple time scales, we construct a Temporal Feature Extraction Block that combines attention mechanisms with multi-scale convolutional layers, enhancing the model’s ability to handle complex traffic pattern changes and capture flow variations and temporal dependencies. Next, we leverage multi-graph adjacency matrix fusion and dynamic Chebyshev graph convolutional networks to capture the spatial dependencies of the traffic network. Experiments on the PeMS04 and PeMS08 datasets show that, compared to conventional methods, the proposed method reduces the Mean Absolute Error (MAE) of traffic flow prediction one hour ahead to 18.33 and 13.72, respectively. The source code for this paper is available at https://github.com/tyut-zjb/TMDCN.

## Introduction

With the continuous improvement of economic conditions, the number of private vehicles has rapidly increased, leading to a growing number and complexity of traffic roads. This, in turn, has exacerbated urban traffic congestion. The development of artificial intelligence (AI) has become crucial for solving traffic-related issues, with traffic flow prediction being a key technology for intelligent transportation systems aimed at alleviating congestion. Traffic flow prediction involves sampling historical traffic data, big data analysis, and mathematical modeling, followed by deep learning techniques to predict traffic conditions for future time periods. Accurate predictions can aid in optimizing route planning, enhancing traffic management, and guiding vehicle dispatching, thereby mitigating traffic congestion to the greatest extent possible^1^.

Traditional statistical models such as ARIMA^2^ and classical machine learning algorithms like SVM^3^, k-NN^4^, and Decision Trees^5^ have been widely applied in early traffic flow prediction tasks. While these methods can capture basic temporal patterns, they often assume data stationarity and rely heavily on manual feature engineering, limiting their ability to handle the nonlinear and complex nature of traffic data, especially in large-scale and high-dimensional scenarios.

Gated Recurrent Units (GRU) and Long Short-Term Memory Networks (LSTM)^6–8^ have proven effective in modeling long-term dependencies in time series. Convolutional Neural Networks (CNN)^9–11^ can capture spatial correlations between different regions. Ma et al.^12^ developed a short-term traffic speed prediction method based on spatiotemporal traffic flow analysis and a combination of deep learning models, as well as a hybrid spatiotemporal feature selection algorithm (STFSA) that combines CNN and GRU.

Zhao et al.^13^ proposed a novel traffic prediction model based on Neural Networks called the Temporal Graph Convolutional Network (T-GCN). This model combines Graph Convolutional Networks (GCN) and Gated Recurrent Units (GRU). The GCN^14^ is used to learn complex topological structures to capture spatial dependencies, while GRU is employed to learn the dynamic changes in traffic data to capture temporal dependencies. T-GCN has made significant improvements in traffic flow prediction due to its comprehensive spatiotemporal modeling ability, 4efficient feature extraction, strong generalization capability, and ability to adapt to various external factors.

The Transformer model^15^, with its core self-attention mechanism, is able to capture long-range dependencies within input sequences, which is crucial for understanding the complex patterns in historical data. The self-attention mechanism helps the model identify traffic patterns for specific time periods (such as holidays or weekdays) and apply them to future predictions^16–19^. Zheng et al.^20^ proposed a Graph Multi-Head Attention Network (GMAN) for predicting traffic conditions at different time steps in road network graphs. GMAN uses an encoder-decoder architecture with multiple spatiotemporal attention blocks to model the influence of spatiotemporal factors on traffic conditions. Although GMAN demonstrates excellent performance in handling spatiotemporal dependencies, it does not fully consider the road network structure and periodic characteristics of traffic flow, limiting its ability to extract spatiotemporal features.

Periodic traffic flow characteristics, such as daily, weekly, and seasonal patterns, are vital for accurate predictions. These patterns reflect dynamic traffic variations, such as peak hours during the morning and evening, low traffic at midnight, and differences between weekdays and weekends. These periodic characteristics reflect the time-varying dynamics of traffic flow, such as high traffic during morning and evening peak hours, low traffic at midnight, differences in traffic flow on weekdays versus weekends, and the impact of specific holidays and large events on traffic flow. If a model fails to capture these periodic features, it may make large prediction errors during peak and off-peak hours, affecting traffic management and scheduling decisions.

Furthermore, improving the self-attention mechanism can enhance the model’s ability to extract spatiotemporal features from both time and space dimensions. Guo et al.^21^ proposed an Attention-based Spatial-Temporal Graph Convolutional Network (ASTGCN), which combines GCN for handling spatial dependencies and Temporal Convolution Networks (TCN) for capturing temporal dependencies. Song et al.^22^ introduced a new Spatiotemporal Graph Convolutional Module (STSGCN) that directly captures local spatiotemporal correlations. They constructed a multi-module layer to capture heterogeneity in the spatiotemporal graph. Jiang et al.^23^ proposed the Propagation Delay-Aware Transformer (PDFormer), which introduces the concept of propagation delay, considering the time delay in information propagation across the road network. This allows the model to more accurately capture the transfer process of traffic flow between road segments. Traditional absolute position encoding may lead to information loss or inaccuracy in long sequences, whereas relative position encoding can better capture the relative distance between elements in the input sequence, thereby effectively handling long-range dependencies. To adapt to dynamic changes, Guo et al.^24^ proposed improving traffic flow prediction performance by learning the dynamics and heterogeneity of spatiotemporal graph data. The model captures temporal variations in the traffic network by introducing a dynamic graph structure and uses a dynamic adjacency matrix to reflect the state of the road network. This dynamic graph structure enables the model to adapt to traffic pattern changes across different time periods, such as the differences between peak and off-peak hours. The model’s heavy reliance on the precise modeling of propagation delay may hinder its generalization to other road networks, as it might not perform well when applied to regions with different traffic patterns.

Fang et al.^25^ introduced a wavelet-based spectral graph attention network that disentangles spatial and temporal features by transforming traffic signals into the wavelet domain. This allows the model to capture multi-frequency traffic patterns with improved efficiency. However, its performance depends on stable spectral decompositions, which may limit adaptability in highly dynamic scenarios. PatchSTG^26^ proposed a scalable transformer framework that partitions the traffic network into spatial patches, enabling efficient modeling of large-scale road networks. It learns both intra- and inter-patch dependencies using a two-stage attention mechanism. While effective for scalability, fixed patch division may reduce the model?s ability to capture fine-grained spatial interactions.

To address the above challenges, we propose a novel model named Temporal Multi-scale Dynamic ChebNet Network (TMDCN), which integrates multi-scale temporal feature extraction, multi-source spatial relationship modeling, and dynamic high-order graph convolution. The key innovations and contributions of this paper are as follows:

Temporal Feature Extraction Block: We design a temporal feature extraction block that combines a self-attention mechanism with multi-scale convolutional layers, enabling the model to capture both short-term fluctuations and long-term trends in traffic flow data.

Multi-Adjacency Matrix Fusion: We propose a novel fusion strategy that integrates three types of adjacency matrices-physical connectivity, similarity-based relationships (via DTW), and dynamic data-driven graphs-capturing complex spatial correlations beyond traditional graph structures.

Dynamic Chebyshev Graph Convolution: We introduce a dynamic ChebNet module that performs high-order graph convolutions with time-varying adjacency matrices, enabling the model to dynamically adapt to traffic pattern changes over time.

Superior Performance and Efficiency: Experiments on real-world datasets (PeMS04 and PeMS08) show that TMDCN outperforms state-of-the-art models in terms of accuracy and efficiency, especially in long-term prediction scenarios.

## Problem formulation

Traffic flow prediction refers to the task of forecasting traffic flow over a future time period based on historical traffic flow data. We represent the road network as a weighted graph $G = (S, E, A)$, where:

- $S$ is the set of traffic sensors, represented as $S = \{ s_1, s_2, \ldots , s_N \}$, where $N$ is the number of sensors. Each sensor $s_i$ is responsible for recording the traffic flow data at its location. The location of the sensor determines the spatial distribution of the traffic data.
- $E$ is the set of edges in the graph, defined as $E = \{ e_{ij} \mid s_i, s_j \in S \}$. Each edge $e_{ij}$ represents a direct connection or spatial relationship between sensor $s_i$ and sensor $s_j$, which may be derived from the physical road topology or spatial proximity. These edges define the topological structure of the sensor network and are used to model spatial dependencies.
- $A \in {\mathbb {R}}^{N \times N}$ is the adjacency matrix, where $A_{ij}$ represents the connection weight between sensor $s_i$ and sensor $s_j$. In the simplest case, if two sensors are directly connected, then $A_{ij} = 1$; otherwise, $A_{ij} = 0$. The adjacency matrix encodes the spatial relationships in the graph and serves as the basis for graph-based modeling.

Traffic flow historical data is collected by the traffic sensor nodes in the road network. The data is recorded at fixed time intervals, capturing the number of vehicles passing through each sensor node during that period. A time step refers to the time unit used for data collection and analysis. In traffic flow prediction, a time step is a fixed and continuous time interval used to record and process traffic data. For example, if the time step is set to 5 minutes, traffic flow data is recorded every 5 minutes. The data recorded by each sensor at time step $t$ can be represented as $X_i^t$, where $i$ is the index of the sensor and $t$ is the time step. The data recorded by all sensors at time step $t$ can be represented as a vector: $X^t = (X_1^t, X_2^t, \ldots , X_N^t) \in {\mathbb {R}}^{N \times 1}$.

**Fig. 1:**
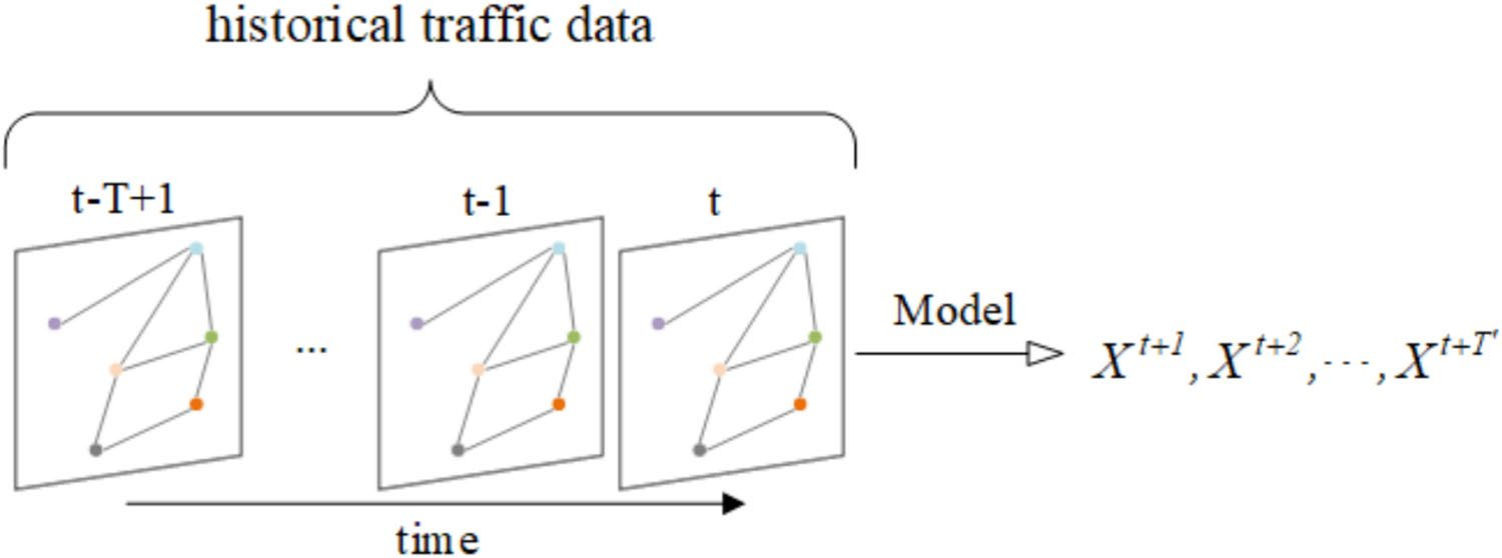
The historical traffic flow data is used by the model to generate predictions for future traffic flow, where the historical data includes both temporal and spatial dimensions.

Given a graph $G$ and the feature matrix for the historical data over $T$ time steps, the goal of traffic flow prediction is to learn a function $f$ that can predict the feature matrix for the next $T'$ time steps, as shown in Fig. 1. This mapping relationship can be expressed as follows:

1 $$\begin{aligned} f(G; X^{t-T+1}, \ldots , X^{t-1}, X^t) \rightarrow X^{t+1}, X^{t+2}, \ldots , X^{t+T'} \end{aligned}$$

## Method

**Fig. 2:**
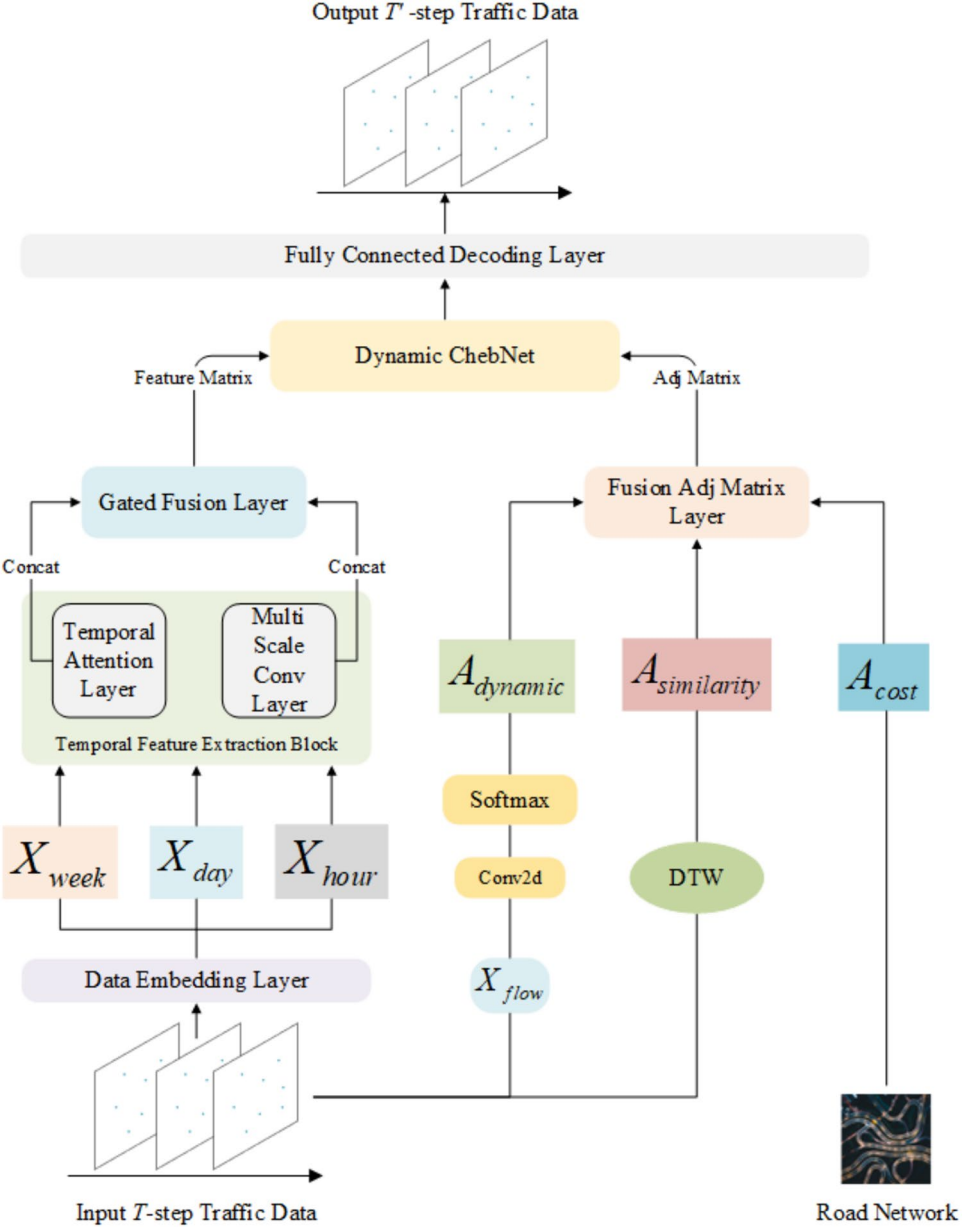
The framework of TMDCN. The model is generally divided into three modules, the temporal feature extraction module, the three-graph fusion adjacency matrix, and the dynamic Chebyshev graph convolution network.

The overall model architecture is shown in Fig. 2. The input data first passes through the Data Embedding layer, which performs feature dimension expansion and temporal sequence embedding. The data is then split by time and passed through multiple layers of self-attention and multi-scale convolutional layers to extract temporal information. The adjacency matrix is derived from the dataset, and a similarity matrix is computed using the DTW^27^ algorithm. A dynamic adjacency matrix is generated based on traffic flow features, and these three adjacency matrices are fused in a fusion layer to extract spatial information. Temporal information serves as the feature matrix, and spatial information is represented as the adjacency matrix, which is passed into the Dynamic ChebNet layer for encoding each sensor node. The Chebyshev graph convolution captures high-order relationships between nodes in the graph. Finally, a fully connected network is used as the decoder to output the predicted results.

The main innovations of the model lie in three modules: the temporal feature extraction module, the three-graph fusion adjacency matrix, and the dynamic Chebyshev graph convolution network.

The temporal feature extraction module consists of two sub-modules: the self-attention mechanism and the multi-scale convolution kernel fusion. The self-attention mechanism is inspired by the encoder part of the Transformer architecture. By calculating the vector similarity between different time periods, it captures the dependencies across time intervals. This mechanism allows the model to flexibly learn the interactions between different time scales, enhancing the model’s ability to perceive temporal dependencies. The multi-scale convolution kernel part performs feature extraction at multiple granularities using convolutional kernels of different sizes. The small-scale kernels focus on short-term traffic flow features, effectively capturing fluctuations in a short time span, while the large-scale kernels focus on long-term features, capturing the long-term trends in traffic flow from a global perspective. These two sub-modules are fused through a gating mechanism, considering both short-term and long-term traffic flow features at different time granularities, thus comprehensively improving the model’s ability to capture dynamic temporal changes and achieving more accurate traffic flow prediction.

The fusion of adjacency matrices is primarily based on three methods. The first is the physical spatial adjacency matrix of the sensors, constructed based on the sensor connection information provided in the dataset. It reflects the physical distance and connectivity between sensors. The second is the similarity adjacency matrix based on the DTW algorithm. Through the DTW algorithm, we observe that although some roads are not directly connected in physical space, their traffic flow patterns exhibit high similarity. These roads, despite their lack of direct physical connections, are highly relevant in traffic flow prediction but are often overlooked by traditional methods. Therefore, we consider these roads to be connected in some way for traffic flow prediction. Lastly, the dynamic adjacency matrix, unlike the static adjacency matrix, is time-varying and captures the dynamic characteristics of traffic flow in real-time. By training on traffic flow data from different time periods, we can obtain these dynamic adjacency matrices, which more accurately reflect the spatiotemporal evolution of traffic flow.

The dynamic Chebyshev graph convolution network combines graph convolution and Chebyshev polynomial approximation methods to efficiently capture high-order spatial relationships between nodes in the graph. In the traffic flow prediction task, the sensor network forms a complex graph structure, where the spatial relationships between nodes exhibit dynamic changes over time. Traditional Graph Convolutional Networks (GCNs) rely on static adjacency matrices, which cannot fully capture the dynamic temporal features present in traffic flow data. The dynamic Chebyshev graph convolution network uses Chebyshev polynomials to approximate the graph Laplacian matrix, reducing the computational complexity of matrix operations during graph convolution. In each convolution layer, the network not only considers the adjacency relations at the current time step but also efficiently captures the influences of distant nodes through the recursive formula of Chebyshev polynomials. In particular, the dynamic Chebyshev graph convolution network can handle time-varying adjacency matrices, enabling it to flexibly adapt to the time-varying nature of traffic flow. By feeding traffic flow data into this network, the model can effectively predict traffic flow while capturing both spatial and temporal dynamics. Compared with static graph convolutional networks, the dynamic Chebyshev graph convolutional network not only improves computational efficiency but also better simulates the complex spatial and temporal dependencies in traffic flow, thereby enhancing prediction accuracy.

### Data embedding layer

The Data Embedding layer is used to expand the feature dimensions of the input data and embed the temporal sequence information. Specifically, the layer consists of two sub-layers: Token Embedding and Positional Encoding, combined with Dropout to improve the model’s generalization ability.

#### Token embedding

First, the input traffic flow data is passed through the TokenEmbedding layer, which performs a linear transformation to map the input data’s feature dimensions to a higher embedding space. The original input data $X \in {\mathbb {R}}^{B \times N \times T \times F}$, where $B$ denotes the batch size, $N$ the number of sensors, $T$ the number of input time steps, and $F$ the number of features, is mapped to a high-dimensional space $X_D \in {\mathbb {R}}^{B \times N \times T \times D}$, where $D$ is the embedding dimension. Subsequently, LayerNorm is applied to normalize the result, improving the stability and efficiency of the training process.

#### Positional encoding

Since the self-attention mechanism lacks positional information and cannot capture the relative position of time steps, a sinusoidal positional encoding is applied to embed temporal positions. Additionally, offsets are used to represent traffic flow at different times. The following formulas, based on the approach described in the Transformer model, are applied to the positional encoding with added offsets:

2 $$\begin{aligned} & PE(pos, 2i) = \sin \left( \frac{pos + \text {offsets}[t]}{10000^{2i / D}}\right) \end{aligned}$$

3 $$\begin{aligned} & PE(pos, 2i + 1) = \cos \left( \frac{pos + \text {offsets}[t]}{10000^{2i / D}}\right) \end{aligned}$$

The formula provides a method to embed the temporal information while also incorporating different time periods of traffic flow. Here:

- $pos$ denotes the absolute position index within the input sequence.
- $i$ denotes the dimension index within the embedding.
- $D$ is the total embedding dimension.
- $\text {offset}(t)$ is a manually or heuristically defined shift that adjusts the base position to distinguish between different temporal sources. Specifically, we set $\text {offset}(t) = 2016$, 288, and 12 for traffic sequences from one week ago, one day ago, and one hour ago, respectively, corresponding to the number of 5-minute intervals within those durations. This offset prevents positional overlap and allows the model to differentiate temporal contexts during learning.
- The constant $10000$ is used as a scaling factor to generate varying wavelengths across different dimensions, ensuring that each dimension learns different periodic representations.

### Temporal feature extraction block

**Fig. 3:**
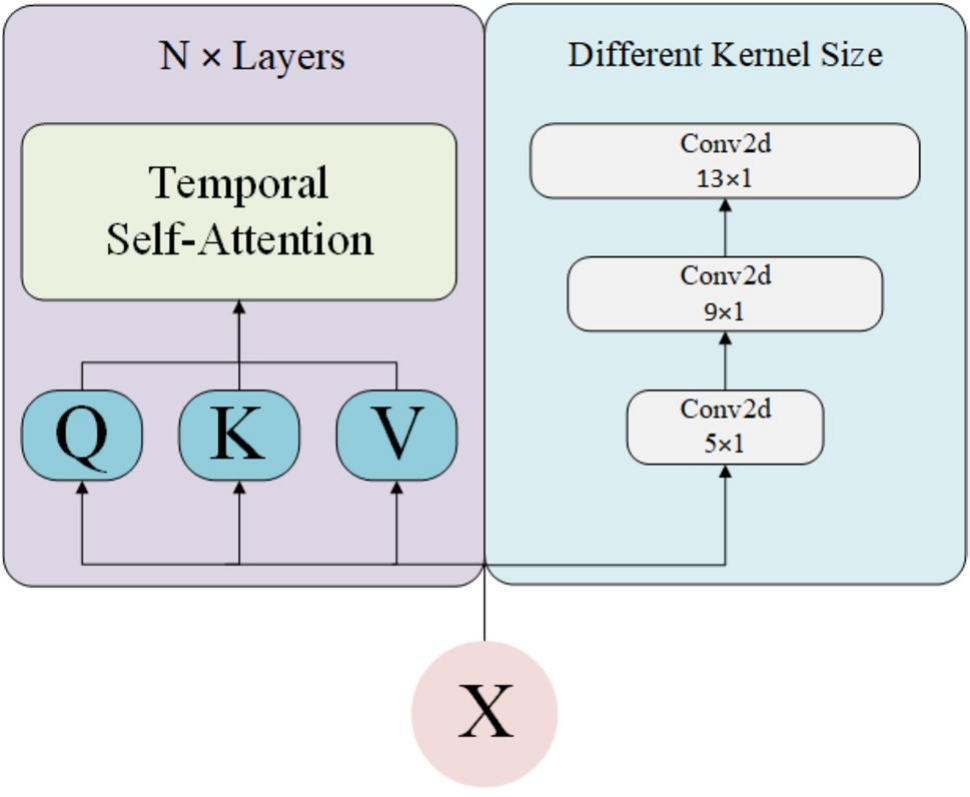
Temporal Feature Extraction Block. On the left, the input passes through N layers of temporal self-attention mechanisms, while on the right, it undergoes a series of 2D convolutional layers with varying kernel sizes.

The structure of the temporal feature extraction module is shown in Fig. 3. First, the data is divided into three parts based on time intervals, which are then passed through multi-layer temporal self-attention layers and multi-scale temporal convolutional layers to extract temporal features. Finally, a gating mechanism is used to effectively fuse the two inputs, thereby extracting the temporal features.

#### Temporal attention layer

Each time step of the input is mapped to three different spaces: Query, Key, and Value. Specifically, given the input matrix $X$, the Query, Key, and Value are obtained through the following linear transformations:

4 $$\begin{aligned} Q = X W_Q, \quad K = X W_K, \quad V = X W_V \end{aligned}$$

Next, the attention scores are computed by taking the inner product of the Query and Key. The Softmax function is applied to obtain the attention weights, and the weighted output is computed by multiplying the attention weights with the Value matrix $V$:

5 $$\begin{aligned} \text {AttentionOutput} = \text {Softmax}\left( \frac{QK^T}{\sqrt{d}} \right) \cdot V \end{aligned}$$

In order to enhance the expressiveness of the network and avoid vanishing gradients, residual connections and Layer Normalization are applied. After each attention module, the output is added to the input $X$ and processed by Layer Normalization. Then, a Feed-Forward Network (FFN) further processes the attention output. The FFN consists of two linear transformations followed by a ReLU activation function. Finally, residual connections and Layer Normalization are applied again to stack multiple Temporal Attention Layers, forming a multi-layer temporal attention network. The output of each layer serves as the input to the next, ultimately yielding the global temporal dependency features of the time series.

#### Multi-scale convolution layer

In order to capture multi-scale features in the time series, the model adopts a multi-scale convolutional structure. The model uses a series of 2D convolutional layers (Conv2d) with different kernel sizes to extract temporal features at different time scales, thereby effectively modeling the dependencies at various temporal scales. During the forward pass, each convolution layer processes the input using a kernel size defined by the input parameter $\texttt {kernel\_sizes}$. The output of each convolution layer is normalized through Layer Normalization to ensure stable training, and residual connections are used to retain the original feature information.

For each convolution layer $i$, the convolution operation is defined as:

6 $$\begin{aligned} \text {Conv}_i(X) = \text {Conv2d}(X, K_i) \end{aligned}$$

Where $K_i$ is the $i$-th convolution kernel with a size of $(k_i, 1)$, and the kernel size $k_i$ is applied to the time dimension $T$, ensuring that features from different time scales are captured.

After all convolution layers have been processed, the model normalizes their outputs through Layer Normalization, and the final output is obtained through the residual connection:

7 $$\begin{aligned} X_{\text {output}} = \text {LayerNorm}(\text {ConvOut}(X) + X) \end{aligned}$$

This multi-scale convolutional time model effectively captures dependencies across different time steps in the sequential data. The varying kernel sizes allow the model to extract features from multiple temporal scales, thereby improving its performance.

### Gated fusion

To effectively combine features extracted from different modules, the model adopts a Gated Fusion mechanism to dynamically adjust the importance of each feature. This module processes the feature matrices from the temporal attention and multi-scale convolution modules separately through two fully connected layers. The Gated Fusion mechanism then combines these features and outputs the fused feature representation.

The two feature matrices are first processed by two linear transformations. The transformed feature matrices are then summed and passed through a Sigmoid activation function to generate the gating function $G$, which indicates the importance of each feature:

8 $$\begin{aligned} G = \text {sigmoid}(TA + MC) \end{aligned}$$

Here, $TA$ represents the feature matrix from the Temporal Attention mechanism, and $MC$ represents the feature matrix from the Multi-Scale Convolution module. The summation of the two transformed feature matrices is passed through the Sigmoid function, which generates the gate $G$ in the range of [0, 1], representing how much weight should be given to the $TA$ and $MC$ features.

The fused features are obtained by applying the gating mechanism to weight the two feature matrices:

9 $$\begin{aligned} X_{\text {output}} = G \cdot TA + (1 - G) \cdot MC \end{aligned}$$

This gating mechanism dynamically adjusts the contribution of the Temporal Attention and Multi-Scale Convolution features, producing the final fused feature representation.

### Multi adjacency matrix fusion

In real-world traffic networks, the deployment of sensors is often sparse and irregular. Constructing an adjacency matrix solely based on whether two sensors are physically connected may lead to overly sparse graphs with disconnected components. To fully utilize the information from different sources of adjacency matrices, this model designs an adjacency matrix fusion module. The module performs weighted fusion of the Cost Adjacency Matrix, Similarity Adjacency Matrix, and Dynamic Adjacency Matrix, generating a final fused adjacency matrix to capture richer spatial relationships.

Each adjacency matrix is of size $N \times N$, where $N$ is the number of sensors. For any element $A_{ij}$ in these matrices, it indicates whether there is a connection between sensor $i$ and sensor $j$ under the corresponding criterion.

#### Cost adjacency matrix

$A_{\text {cost}}$ represents the inherent spatial structure of the road network in traffic flow data. It is expressed as an adjacency matrix and can be derived from the adjacency relationships between nodes in the dataset. The matrix is defined as follows:

10 $$\begin{aligned} A^{\text {cost}}_{ij} = {\left\{ \begin{array}{ll} 1, & \text {if } i \text { and } j \text { are physically connected} \\ 0, & \text {otherwise} \end{array}\right. } \end{aligned}$$

#### Similarity adjacency matrix

**Fig. 4:**
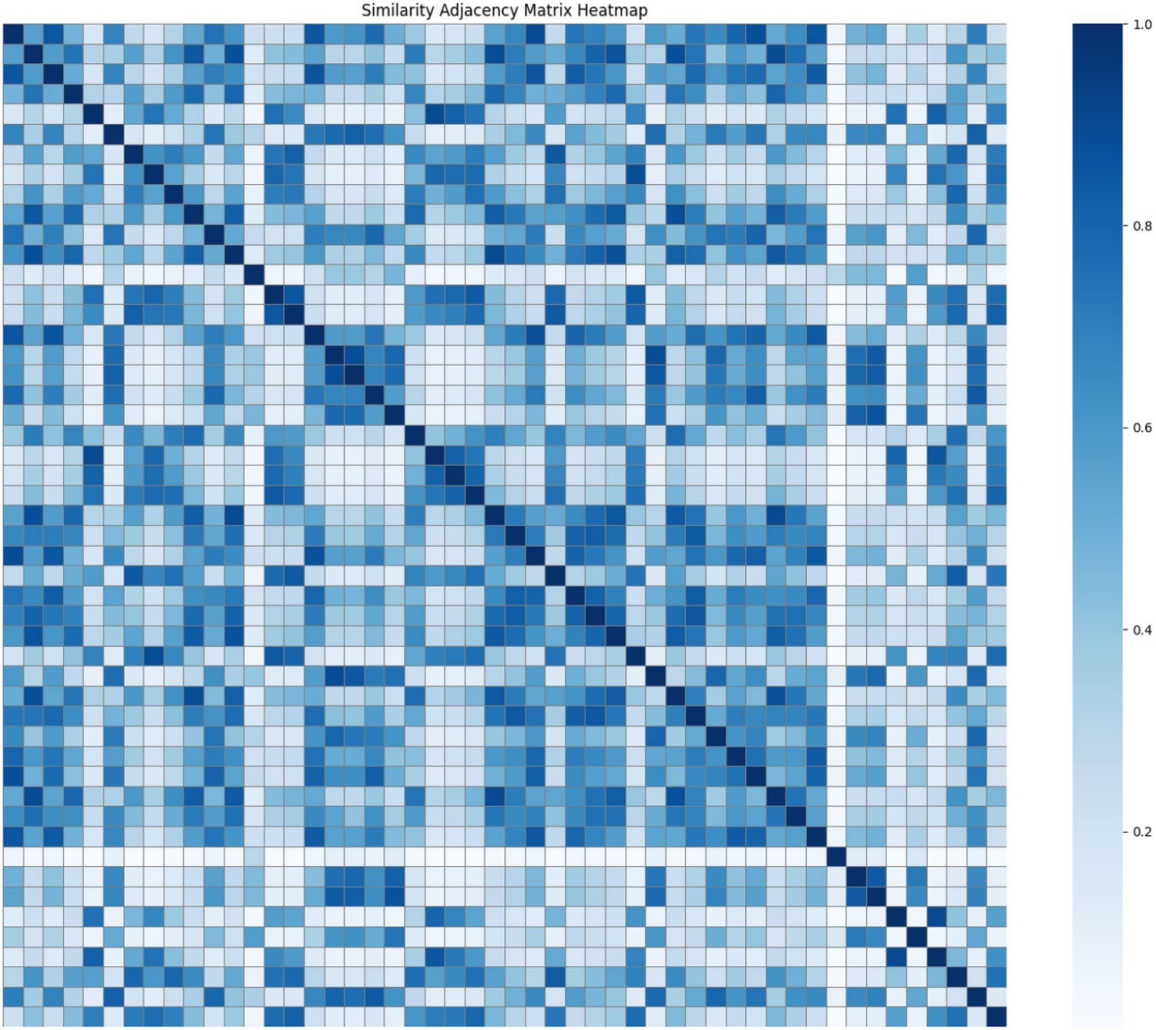
The heatmap reflects the similarity between different sensors. Darker colors indicate higher similarity between the two, suggesting that they exhibit more similar trends over different time periods.

$A_{\text {similarity}}$ represents the similarity between roads based on the trend and pattern of traffic flow changes over time. The Euclidean distance may not effectively measure the similarity between two time series that exhibit similar shapes but asynchronous temporal patterns. Two different roads may show similar change trends during different time periods, and DTW can effectively address this issue. The heatmap of the top 50 sensor similarities is shown in Fig. 4. The matrix is defined as:

11 $$\begin{aligned} A^{\text {similarity}}_{ij} = \exp \left( -\text {DTW}(i, j)\right) \end{aligned}$$

#### Dynamic adjacency matrix

$A_{\text {dynamic}}$ represents the dynamic adjacency matrix, which is used to dynamically generate the adjacency relationships between sensor nodes based on traffic flow data. The basic idea is to extract features from the input traffic flow data through convolution operations and calculate a new dynamic adjacency matrix via matrix operations to represent the spatial relationships between different time steps. The first feature, traffic flow $X_{\text {flow}} \in {\mathbb {R}}^{B \times N \times T}$, is used, and the data dimensions are transformed into $X_{\text {input}} \in {\mathbb {R}}^{B \times C \times N}$, where $C$ is the number of channels output by the convolution layer. The dynamic adjacency matrix layer is defined as follows:

12 $$\begin{aligned} A_{\text {dynamic}} = \text {Softmax}\left( \text {ReLU}\left( X_{\text {input}}^T \cdot W_{\phi }^T \cdot W_{\phi } \cdot X_{\text {input}}\right) \right) \end{aligned}$$

In Eq. (12), we generate a dynamic adjacency matrix from the input node features using a symmetric bilinear transformation. Specifically, we first compute a similarity matrix $S = X_{\text {input}}^T W_{\phi }^T W_{\phi } X_{\text {input}}$, where $W_{\phi } \in {\mathbb {R}}^{d \times d}$ is a trainable weight matrix. This symmetric form ensures that $S \in {\mathbb {R}}^{N \times N}$ encodes mutual similarity between nodes in a learnable manner.

A ReLU activation is applied to remove negative similarity values, and the Softmax function is applied row-wise to normalize the edge weights. The resulting matrix $A_{\text {dynamic}}$ is thus non-negative and row-stochastic, where each row can be interpreted as an attention distribution over neighboring nodes. This structure enables the model to dynamically learn meaningful and adaptive spatial dependencies from traffic data, and the parameters $W_{\phi }$ are optimized end-to-end during training via backpropagation.

#### Fusion adjacency matrix

To integrate the three adjacency matrices mentioned above, this method designs a weighted fusion mechanism, where the weights are controlled by three learnable parameters $a$, $b$, and $c$, which determine the contribution of each adjacency matrix in the fusion process. The weighted sum of the three adjacency matrices is computed as follows to obtain the final fused adjacency matrix $A_{\text {fusion}}$:

13 $$\begin{aligned} A^{\text {fusion}}_{ij} = {\left\{ \begin{array}{ll} 1, & \text {if } a \cdot A^{\text {cost}}_{ij} + b \cdot A^{\text {dynamic}}_{ij} + c \cdot A^{\text {similarity}}_{ij} > q \\ 0, & \text {otherwise} \end{array}\right. } \end{aligned}$$

The fusion weights $a$, $b$, and $c$ are learnable parameters that control the contribution of the cost, dynamic, and similarity-based adjacency matrices in the final fused adjacency matrix. These weights are initialized to $1.0$ using standard initialization in PyTorch, ensuring that each adjacency matrix is initially weighted equally. The parameters are then optimized during training using backpropagation.

Where $q$ is a threshold value that determines the connection strength.

### Dynamic ChebNet

In order to more effectively capture the spatial dependencies between sensor nodes in the graph, the model introduces the Dynamic Chebyshev Graph Convolution Network (Dynamic ChebNet), which performs graph convolution based on Chebyshev polynomials. This method can efficiently handle higher-order relationships between nodes in the graph structure. The output data from the temporal feature extraction block is transformed to $X \in {\mathbb {R}}^{B \times N \times T \times D}$, where each node’s feature matrix is obtained. The adjacency matrix aggregation block outputs the fused adjacency matrix $A \in {\mathbb {R}}^{B \times N \times N}$, which serves as the adjacency matrix for the Dynamic ChebNet module.

The Dynamic Chebyshev Graph Convolution Network employs Chebyshev Convolution (ChebConv) layers to extract spatial information. The convolution operation for each layer can be expressed as:

14 $$\begin{aligned} X^{(l+1)} = \sum _{k=0}^{K-1} {\hat{T}}_k(A) X^{(l)} W_k \end{aligned}$$

- $X^{(l)}$ is the node feature matrix at the $l$-th layer.
- ${\hat{T}}_k(A)$ is the $k$-th order Chebyshev polynomial applied to the adjacency matrix $A$ for convolution.
- $W_k$ is the weight of the $k$-th order Chebyshev convolution kernel.
- $K$ is the order of the Chebyshev polynomial.

In Eq. (14), we use Chebyshev polynomial approximation with $K = 3$, which has been shown to balance computational efficiency and model expressiveness. A higher value of $K$ could capture more complex spatial dependencies in the graph, but this comes at the cost of increased computational complexity. Specifically, the computational cost of Chebyshev convolution increases linearly with $K$, making higher-order polynomials computationally expensive, especially when scaling to larger graphs.

In each layer, the Chebyshev convolution combines higher-order information from the adjacency matrix, learning the spatial relationships of nodes through multiple convolution operations in the graph.

### Fully connected decoding layer

The fully connected output layer is used to generate the final prediction of the model. This layer first applies layer normalization to the input, followed by processing through multiple fully connected layers. After each fully connected layer, a ReLU activation function is used to increase the model’s ability to express non-linear relationships. The final output is obtained through a linear transformation.

The output of the Dynamic ChebNet is $X_{\text {gcn}} \in {\mathbb {R}}^{B \times N \times D_{\text {gcn}}}$, which is passed to the decoder. After several linear transformations, the output dimension becomes $X_{\text {output}} \in {\mathbb {R}}^{B \times N \times T}$, representing the final prediction of the model. The overall formula of the model is given as follows:

15 $$\begin{aligned} X_i = \text {ReLU}(W_i X_{i-1} + b_i) \end{aligned}$$

## Experiments

### Dataset

To validate the effectiveness of the proposed model, we used two widely recognized real-world traffic flow datasets: PeMS04 and PeMS08. The PeMS (Performance Measurement System) dataset^28^ is obtained from traffic flow detectors on highways in California, developed by the California Department of Transportation. The PeMS dataset includes traffic flow data from multiple sensors distributed across various highways. The PeMS04 and PeMS08 datasets represent traffic data from different regions:

- **PeMS04**: Collected data from 307 sensors at 5-minute intervals, covering traffic flow data for 59 days.
- **PeMS08**: Collected data from 170 detectors at 5-minute intervals, covering traffic flow data for 62 days.

Each detector collects data with three feature dimensions: flow, average speed, and average occupancy. Detailed information about the datasets is shown in Table 1. The datasets are divided into training, validation, and test sets using a 6:2:2 ratio.

**Table 1:** Specific data of the datasets.

Datasets	Nodes	Edges	Data Length	Feature Count	Sample Interval
PeMS04	307	340	16992	3	5min
PeMS08	170	295	17856	3	5min

### Preprocessing

**Fig. 5:**
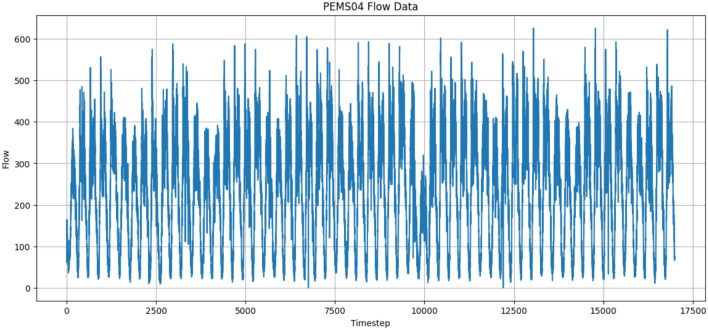
Sensor flow data. This figure shows the traffic flow characteristics of the first sensor in the PeMS04 dataset.

For example, the traffic flow feature data of the first sensor in the PeMS04 dataset exhibits periodicity and large amplitude fluctuations, as shown in Fig. 5.

Therefore, we adopt the data preprocessing method mentioned in ASTGCN to extract time-series features from the data. Specifically, we extract the traffic flow data from one week ago, one day ago, and one hour ago as features to predict the traffic flow one hour ahead. The dataset is divided into training, validation, and test sets with proportions of 60%, 20%, and 20%, respectively. To mitigate the impact of amplitude differences, we apply the Z-score normalization method to the raw data, as shown in the following formula:

16 $$\begin{aligned} Z_{score}(X) = \frac{X - X_{mean}}{X_{std}} \end{aligned}$$

### Experimental setting

The experiments were conducted using an RTX 3090GPU for training, based on Ubuntu 20.04, with Pytorch 2.0.0 and Python 3.8 for implementing the TMDCN model. The prediction target is to forecast the traffic flow for the next hour based on historical traffic flow data from one hour ago, one day ago, and one week ago. The model’s loss function is HuberLoss, the optimizer used is AdamW, and the learning rate scheduler is CosineLRScheduler. The model parameters are shown in Table 2.

**Table 2:** Model structure parameters.

Parameter	Value
learning rate	0.001
weight decay	0.001
batch size	8
epoch	100
dropout	0.2
embed dim	32
attention num layer	4
attention hidden dim	128
multi scale conv unit	[5, 9, 13]
dynamic adj matrix conv out channel	32
dynamic chebnet num layer	2
dynamic chebnet K	3
dynamic chebnet hidden dim	512
dynamic chebnet output dim	256
fc hidden unit	[128, 64, 16]

### Evaluation metrics

In order to analyze the prediction results and evaluate the model’s performance, three metrics were selected for the experiment: Mean Absolute Error (MAE), Mean Absolute Percentage Error (MAPE) and Root Mean Square Error (RMSE). The calculation formulas for these metrics are as follows:

17 $$\begin{aligned} & \text {MAE} = \frac{1}{m} \sum _{i=1}^{m} |Y_i - {\hat{Y}}_i| \end{aligned}$$

18 $$\begin{aligned} & \text {MAPE} = \frac{1}{m} \sum _{i=1}^{m} \frac{|Y_i - {\hat{Y}}_i|}{Y_i} \times 100\% \end{aligned}$$

19 $$\begin{aligned} & \text {RMSE} = \sqrt{\frac{1}{m} \sum _{i=1}^{m} (Y_i - {\hat{Y}}_i)^2} \end{aligned}$$

### Baseline

We compared the TMDCN model with traditional statistical-based methods and recently proposed GNN-based traffic prediction models, which reflect the latest advancements in the field. The baseline models are described as follows:

- **DCRNN**^29^ : A model based on GNN and RNN that combines GRU with bidirectional diffusion convolution.
- **STGCN**^30^ : A model based on GNN and CNN that combines graph convolution with 1D convolution.
- **T-GCN**^13^ : A model that directly computes the update of gated units through graph convolution, enabling spatiotemporal dependency modeling of graph-structured data by deeply integrating GCN and GRU.
- **GWNET**^31^ : A model based on GNN and CNN that combines diffusion graph convolution with gated 1D dilated convolution, and proposes an adaptive adjacency matrix.
- **GMAN**^20^ : A model based on attention mechanisms with spatial, temporal, and transition attention.
- **MTGNN**^32^ : A model based on GNN and CNN that employs adaptive graphs, hybrid jump propagation layers, and dilated inception layers to capture spatiotemporal dependencies.
- **LightCTS**^33^ : A lightweight framework designed for forecasting correlated time series by efficiently modeling inter-series dependencies using a combination of convolutional and attention mechanisms, while maintaining low computational complexity.
- **ASTGNN**^24^ : A model based on convolutional self-attention mechanisms that captures local traffic flow trends through 1D convolutions.
- **DDGCRN**^34^ : A model that decomposes traffic data into spatial and temporal components, utilizing dynamic graph convolution to capture spatial dependencies and recurrent networks to model temporal dynamics for accurate traffic flow prediction.
- **STID**^35^ : A simple yet effective baseline that introduces spatial-temporal identity representations to enhance multivariate time series forecasting by modeling location-specific temporal patterns and enabling efficient spatial aggregation.
- **PDFormer**^23^ : A model based on spatiotemporal self-attention mechanisms, designed with a spatial self-attention module that models local geographical neighborhoods and global semantic neighborhoods through different graph masking methods. Additionally, it introduces a traffic delay-aware feature transformation module that explicitly models temporal delays in the propagation of spatial information.

### Experimental results

The experimental results of the TMDCN model proposed in this paper and the comparison models are shown in Table 3. TMDCN outperforms the baseline models in all three key metrics across both datasets. Specifically, compared to the best baseline model, PDFormer, the MAE of TMDCN decreased by 1.02%, MAPE decreased by 1.68%, and RMSE decreased by 2.46%. The significant reduction in RMSE indicates that the overall deviation of the predicted values is smaller, with a lower probability of outliers, and the predictions closely follow the curve. This demonstrates that the TMDCN model provides better prediction accuracy than the baseline models.

**Table 3:** Performance comparison of different models.

Baseline	PeMS04			PeMS08		
	MAE	MAPE (%)	RMSE	MAE	MAPE/%	RMSE
DCRNN	22.75	14.84	36.83	18.30	11.35	28.52
STGCN	21.82	13.91	34.91	17.87	11.21	27.37
T-GCN	20.95	13.80	33.37	16.55	11.08	25.73
GWNET	19.45	13.44	31.75	15.53	10.61	24.85
GMAN	19.16	13.21	31.62	15.44	10.03	24.59
MTGNN	19.08	12.95	31.46	15.38	10.17	24.93
LightCTS	18.81	12.82	31.21	15.13	9.85	24.43
ASTGNN	18.65	12.63	31.04	14.98	9.62	24.89
DDGCRN	18.59	12.51	30.64	14.67	9.45	23.75
STID	18.55	12.49	29.83	14.22	9.42	23.53
PDFormer	18.52	12.46	29.97	13.85	9.41	23.22
TMDCN	18.33	12.25	29.23	13.72	9.37	22.81

**Fig. 6:**
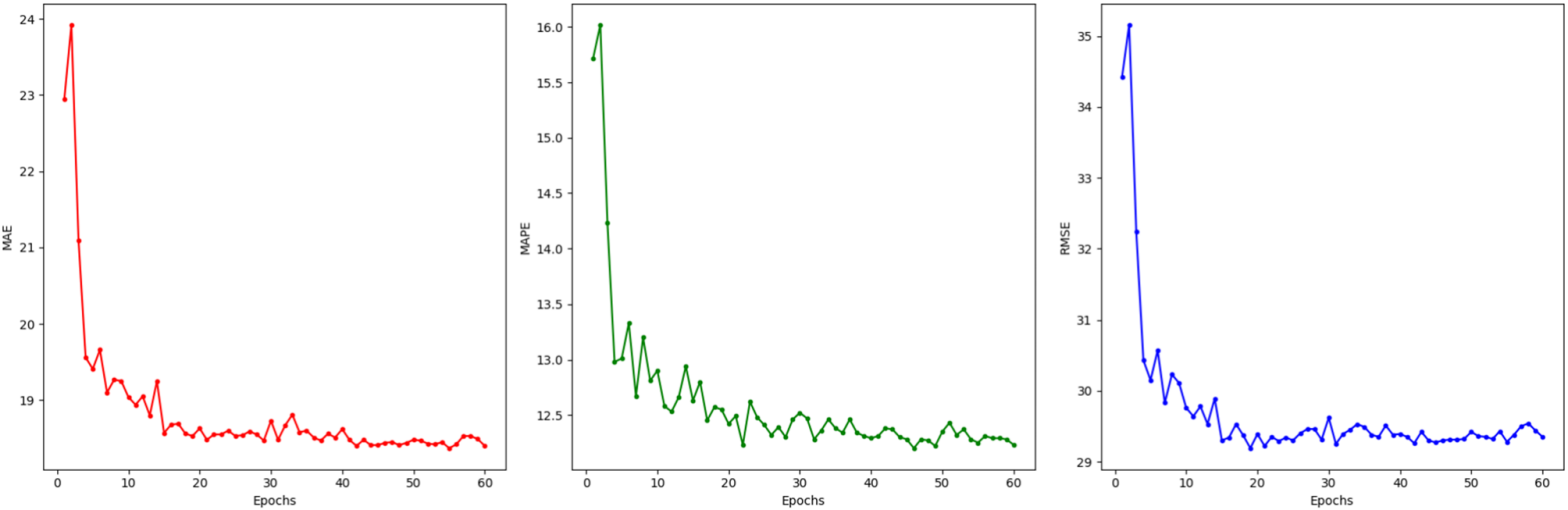
Training process. The left panel shows the MAE, the middle panel displays the MAPE, and the right panel presents the RMSE across training epochs.

The training process is shown in Fig. 6, which presents the changes in three different error metrics (MAE, MAPE, and RMSE) as training progresses. The left plot shows how the Mean Absolute Error (MAE) changes with the number of training epochs, the middle plot shows the Mean Absolute Percentage Error (MAPE) variation, and the right plot shows the Root Mean Square Error (RMSE) over time. As observed, all three metrics are initially high, but they gradually decrease and stabilize as training progresses, indicating that the model is gradually converging through continuous optimization and adjustments. Especially in the early stages of training, the model extracts effective features from the data, which leads to a steady reduction in error.

Since the model reaches a stable state early in the training process, we utilized a learning rate scheduler to dynamically adjust the learning rate in order to avoid poor training results due to excessively large or small learning rates. By reducing the learning rate at appropriate times, the model’s fine-tuning process is further enhanced, improving convergence speed and preventing premature convergence to local minima. Ultimately, the learning rate adjustment allows the model to achieve lower error while maintaining high generalization ability, which effectively enhances prediction accuracy and robustness.

**Fig. 7:**
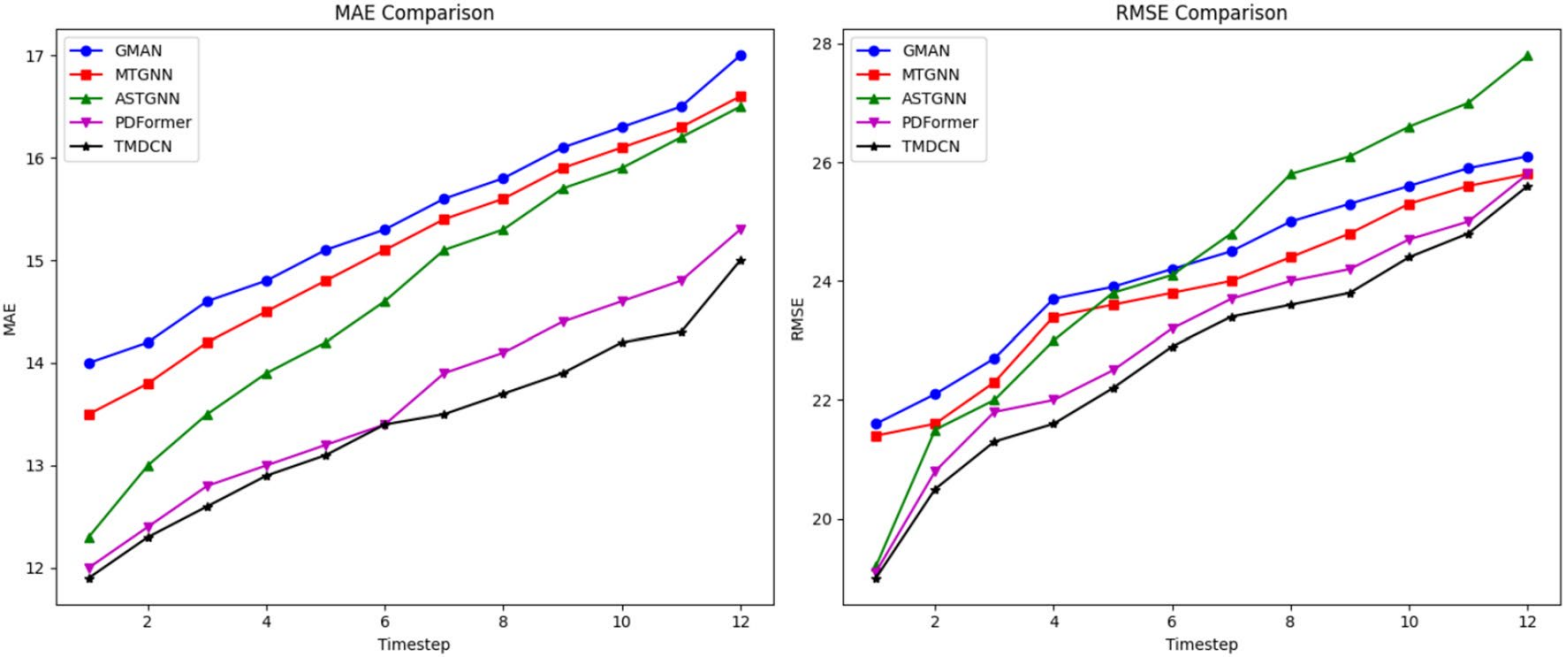
Comparison of multi-step prediction performance of different models on the PeMS08 dataset.

To further compare and analyze the differences in the long-term and short-term prediction performance across models, four representative baseline models were selected, and the MAE and RMSE error variation curves at different prediction time steps were plotted, as shown in Fig. 7. The data presented in the figure indicate the changes in prediction performance as the prediction horizon increases. In general, as the prediction horizon becomes longer, the difficulty of prediction increases, leading to a corresponding increase in prediction error. The results show that TMDCN performs comparably to baseline models in short-term predictions, but significantly outperforms them in long-term predictions.

**Fig. 8:**
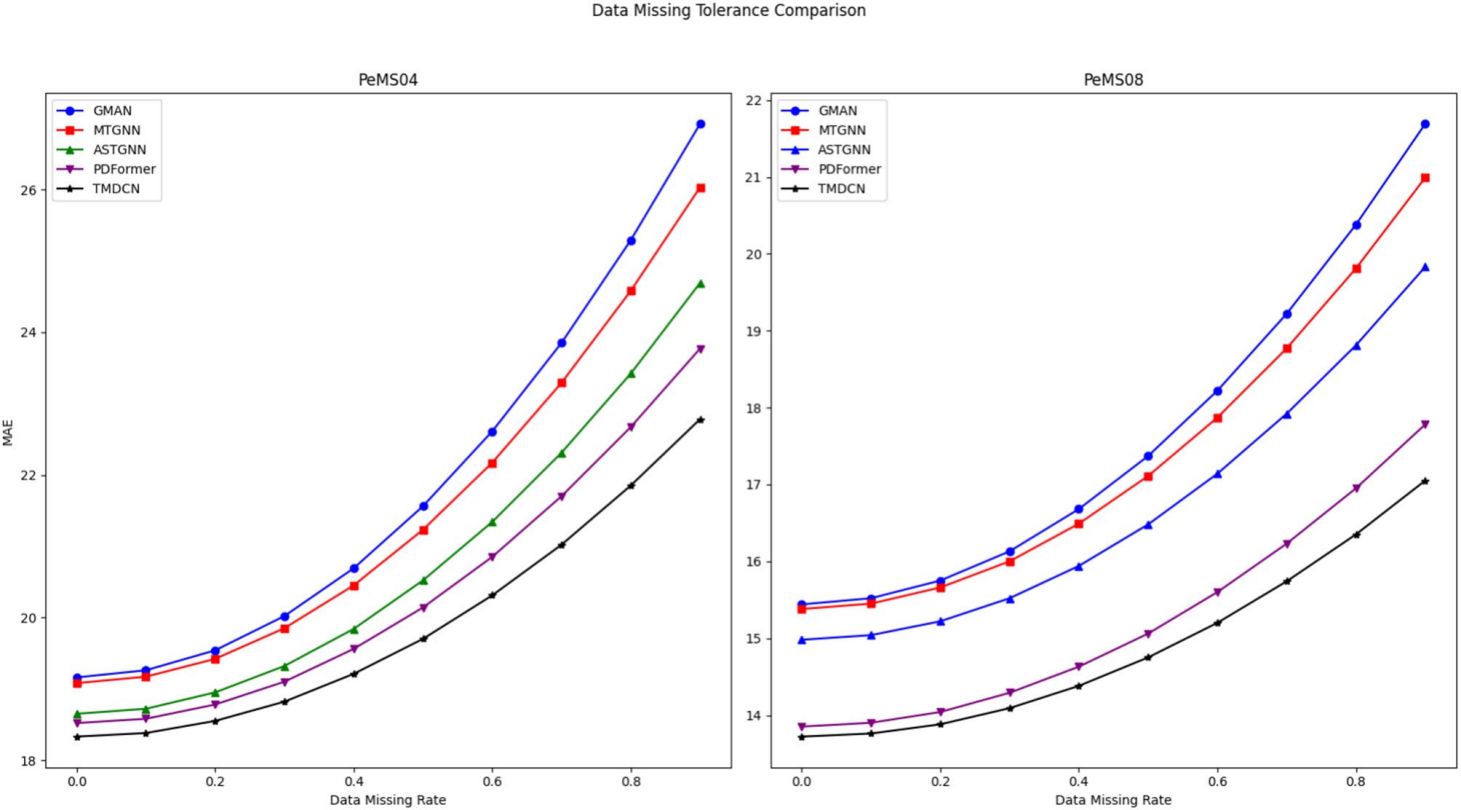
Data missing tolerance comparison under different missing rates. TMDCN consistently outperforms baseline models as the missing rate increases, demonstrating stronger robustness.

In real-world traffic data collection, data missing is a common issue caused by sensor failures, signal interference, and other factors. Therefore, the tolerance of a prediction model to missing data is crucial for its practical applicability. To evaluate the robustness of the proposed model, we randomly remove a certain proportion (ranging from 10% to 90%) of historical traffic data and compare the performance of TMDCN with several state-of-the-art models, with a fixed prediction time window of 1 hour. As shown in Fig. 8, TMDCN consistently maintains stable and superior prediction performance under high missing data rates. Particularly when the missing ratio exceeds 40%, TMDCN demonstrates significantly better tolerance than the baselines. These findings indicate that TMDCN is capable of effectively capturing global spatiotemporal patterns even with incomplete data, highlighting its strong robustness and practical value^36^.

### Ablation study

To verify the effectiveness of different layers in TMDCN, we replace or remove some components based on the original model to form the following variant models:

- w/o Dynamic ChebNet: Replacing Dynamic ChebNet with GCN.
- w/o Multi Scale Conv Layer: Removing the multi-scale convolution layer.
- w/o Dynamic Adjacency Matrix: Removing the dynamic adjacency matrix.
- w/o Gated Fusion: Using simple addition instead of the gated fusion mechanism.

**Fig. 9:**
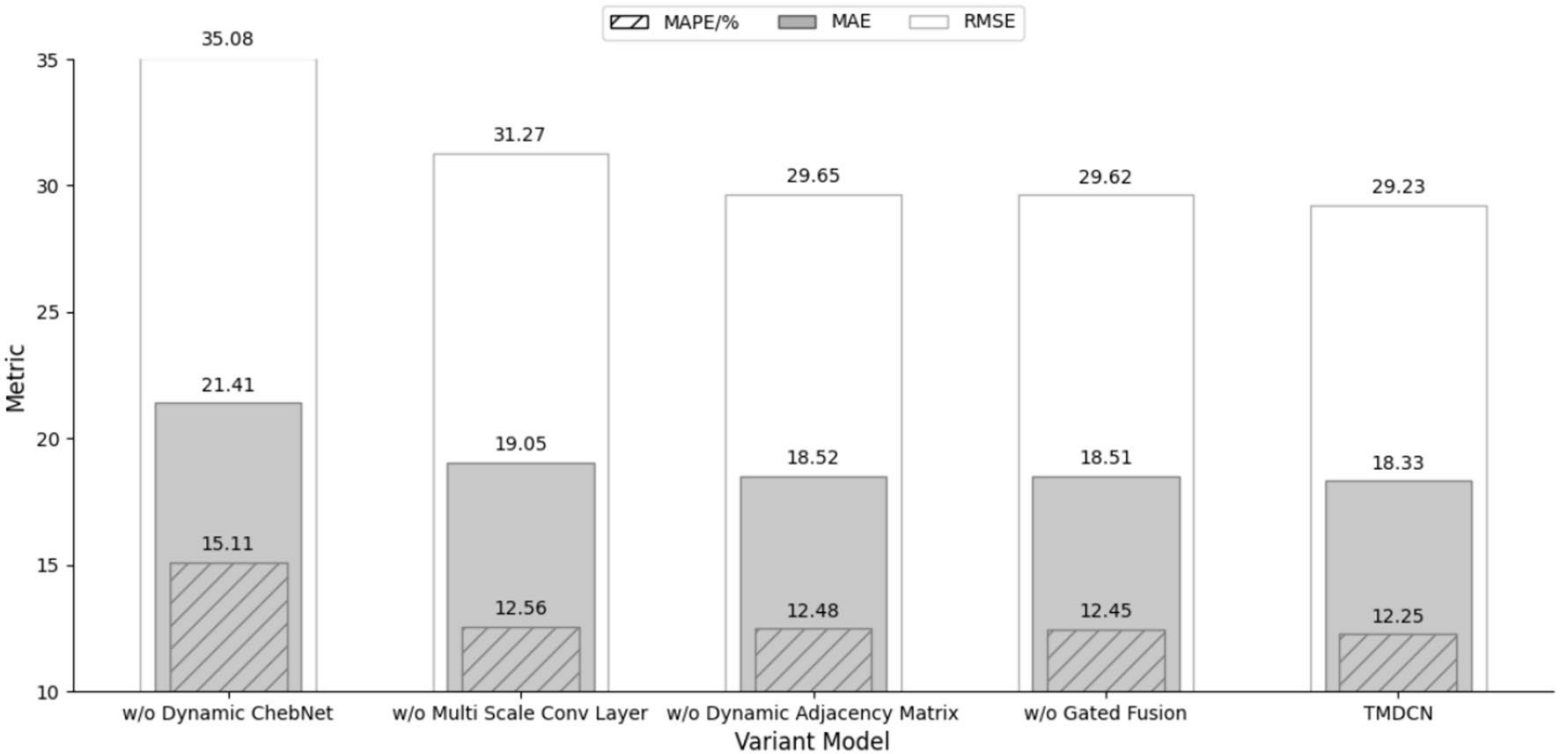
Ablation Study. The metrics shown include RMSE, MAE, and MAPE (indicated by striped bars). Each bar represents the metric values of the respective variant models and the complete TMDCN model.

The ablation experimental results of these variants on the PeMS04 dataset are shown in Fig. 9. As can be seen from the results, replacing the gated fusion mechanism with simple addition causes a 0.18 increase in MAE, indicating that the gated mechanism effectively enhances the model’s ability to fuse information from different time scales, thereby improving prediction accuracy. Moreover, removing the dynamic adjacency matrix leads to a 0.19 increase in MAE, suggesting that the dynamic adjacency matrix captures the spatiotemporal dependencies of traffic flow changes over time, further improving the model’s performance in dynamic traffic environments. Removing the multi-scale convolution layer results in a 0.72 increase in MAE, showing that the multi-scale convolution layer better extracts traffic flow features at different time scales. Replacing ChebNet with GCN causes a significant 3.08 increase in MAE, indicating that ChebNet has a stronger expressive ability than GCN in modeling graph structure information, enabling better capture of high-order spatiotemporal dependencies in traffic flow data. These ablation results further validate the importance of each component in improving model performance.

### Performance comparison

To validate the performance of the model, we compare TMDCN with three other baseline models. The overall structures of GMAN and ASTGNN are time-consuming encoder-decoder structures, while PDFormer and TMDCN both use fully connected networks as decoders. The training time and inference time of the models on the PeMS04 dataset are shown in Table 4. The results indicate that TMDCN, compared to the best-performing PDFormer, reduces training time by 9.53% and inference time by 4.37%.

**Table 4:** Performance comparison of different models.

Baseline	Train time/s	Infer time/s
GMAN	502.34	37.43
ASTGNN	202.15	65.38
PDFormer	127.59	10.29
TMDCN	115.43	9.84

## Conclusion

This study presents a model, TMDCN, based on multi-scale temporal feature extraction and multi-adjacency matrix fusion. By extracting features from historical data and deeply integrating temporal and spatial information, the model effectively captures the spatiotemporal variation patterns of traffic flow and demonstrates superior performance across various traffic flow prediction tasks.

First, the model extends the feature dimensions of the raw data through a data embedding layer and introduces positional encoding to preserve temporal order information. Then, the model uses a multi-scale convolution model to extract traffic flow features at different time scales, further improving its ability to express complex traffic patterns. To capture the spatial dependencies of traffic flow, we construct a fused adjacency matrix based on the cost adjacency matrix, similarity adjacency matrix, and dynamic adjacency matrix, and model higher-order spatial information using Chebyshev Graph Convolution Networks. Finally, the model decodes the traffic flow predictions for future time steps through a fully connected output layer.

In the experimental section, the model outperforms traditional statistical and machine learning-based traffic flow prediction methods on the PEMS04 and PEMS08 datasets. Especially in handling complex spatiotemporal features, the model effectively captures the spatiotemporal correlations of traffic flow changes through dynamic adjacency matrices and multi-layer attention mechanisms, significantly improving prediction accuracy.

Although the proposed TMDCN model demonstrates superior performance in traffic flow prediction, several limitations remain:

Computational Complexity: The integration of multi-scale convolution and dynamic adjacency matrices increases computational overhead, making real-time processing challenging, especially for large-scale traffic networks. Future research can explore more efficient model structures or lightweight versions to enhance real-time applicability.

External Factors: The model primarily focuses on historical traffic flow data without explicitly considering external factors such as weather conditions, road accidents, or special events. In future work, we plan to incorporate external contextual factors (weather, accidents, holidays) into the prediction framework. These can be encoded as auxiliary embeddings and fused with traffic features to further enhance the model?s accuracy and generalizability.

Long-Term Prediction: The model performs well on short-term traffic flow forecasting but may struggle with long-term predictions due to accumulated errors. Future research could investigate hybrid architectures that combine recurrent structures or sequence-to-sequence models for better long-term forecasting.

In future work, we plan to address these limitations by optimizing the model’s computational efficiency, enhancing its generalizability, incorporating external factors, and improving its ability to capture long-term dependencies. Additionally, exploring real-world deployment scenarios and integrating the model into intelligent transportation systems will be an important direction for practical applications.

## Data Availability

The datasets analyzed in this study, PeMS04 and PeMS08, are publicly available at the California Department of Transportation Performance Measurement System (https://pems.dot.ca.gov).

## Code availability

The implementation code for the proposed model is publicly available at GitHub (https://github.com/tyut-zjb/TMDCN) under the MIT License.
